# Role of Synchronous, Moderated, and Anonymous Peer Support Chats on Reducing Momentary Loneliness in Older Adults: Retrospective Observational Study

**DOI:** 10.2196/59501

**Published:** 2024-10-25

**Authors:** Zara Dana, Harpreet Nagra, Kimberly Kilby

**Affiliations:** 1 Supportiv Berkeley, CA United States

**Keywords:** digital peer support, social loneliness, chat-based interactions, older adults

## Abstract

**Background:**

Older adults have a high rate of loneliness, which contributes to increased psychosocial risk, medical morbidity, and mortality. Digital emotional support interventions provide a convenient and rapid avenue for additional support. Digital peer support interventions for emotional struggles contrast the usual provider-based clinical care models because they offer more accessible, direct support for empowerment, highlighting the users’ autonomy, competence, and relatedness.

**Objective:**

This study aims to examine a novel anonymous and synchronous peer-to-peer digital chat service facilitated by trained human moderators. The experience of a cohort of 699 adults aged ≥65 years was analyzed to determine (1) if participation, alone, led to measurable aggregate change in momentary loneliness and optimism and (2) the impact of peers on momentary loneliness and optimism.

**Methods:**

Participants were each prompted with a single question: “What’s your struggle?” Using a proprietary artificial intelligence model, the free-text response automatched the respondent based on their self-expressed emotional struggle to peers and a chat moderator. Exchanged messages were analyzed to quantitatively measure the change in momentary loneliness and optimism using a third-party, public, natural language processing model (GPT-4 [OpenAI]). The sentiment change analysis was initially performed at the individual level and then averaged across all users with similar emotion types to produce a statistically significant (*P*<.05) collective trend per emotion. To evaluate the peer impact on momentary loneliness and optimism, we performed propensity matching to align the moderator+single user and moderator+small group chat cohorts and then compare the emotion trends between the matched cohorts.

**Results:**

Loneliness and optimism trends significantly improved after 8 (*P*=.02) to 9 minutes (*P*=.03) into the chat. We observed a significant improvement in the momentary loneliness and optimism trends between the moderator+small group compared to the moderator+single user chat cohort after 19 (*P*=.049) and 21 minutes (*P*=.04) for optimism and loneliness, respectively.

**Conclusions:**

Chat-based peer support may be a viable intervention to help address momentary loneliness in older adults and present an alternative to traditional care. The promising results support the need for further study to expand the evidence for such cost-effective options.

## Introduction

### Background

The older adult population continues to increase, with global projections of 1 in 6 people being aged ≥60 years by 2030 and estimated increases from 1 billion in 2020 to 1.4 billion in 2030 to 2.1 billion older adults in 2050 [1]. High projections indicate a need to address the health needs of this growing community, in particular the issues related to loneliness and isolation. Notably, as cited in the US Surgeon’s 2023 Advisory titled “Our Epidemic of Loneliness and Isolation” [2], loneliness, isolation, and social connection decrease self-care capacity and quality of life, increase mental and physical risks for depression and cardiovascular diseases, and impose significant financial drain on individual persons’ and the US health care system [3]. For example, due to these factors, Medicare’s spending alone is US $6.7 billion annually [3]. Furthermore, it is well known that, compared to nonsocially isolated persons, socially isolated persons tend to have higher health care costs, marking this a true *public health crisis* [3].

### Loneliness and Optimism

Older persons (aged ≥65 years) may be at higher risk of feeling lonely due to social role changes (eg, grown children moving away from parental home—empty nest and employment retirement), housing shifts (eg, moving into residential care), reduced social networks (eg, widowhood and death of intimate friends of similar ages), the onset of health concerns paired with reduced physical ability, and the loss of independent transportation, which reduces outside social activity [4-6]. Reduced social activity, in turn, may increase feelings of loneliness and social isolation. Loneliness has been defined as a multidimensional concept; broadly, it has been defined by Weiss [7] as a personal feeling that arises at a certain time in life and can affect anyone regardless of age, sex, socioeconomic status, or unique personality traits. More specifically, loneliness may fall into 2 distinct categories, including emotional versus social loneliness [8]. Emotional loneliness refers to the lack of an attachment figure and potential lack of intimacy, which may reinforce feelings of emptiness or abandonment [8]. Social loneliness, by contrast, refers to the lack of a social network to create a sense of belonging or community [8], which may raise feelings of shame, desperation, sadness, or frustration. Loneliness also has an associated stigma in that it is perceived as rooted in weakness or self-pity, meaning that the person ought to eliminate this feeling because it is not a physical ailment. Effective interventions to address the multidimensional layers of loneliness remain limited. This study will focus on an intervention designed to impact social loneliness, specifically the impact of momentary and time-bound peer engagement.

Concurrently, a protective subjective trait to counter the negative impact of loneliness is optimism [9]. Optimism is defined as a personality trait whereby the individual sustains a positive future outlook and expectations [10]. One study with older adults concluded that interventions should focus on fostering a sense of community to enhance optimism and reduce loneliness among this population to improve subjective well-being [11]. Loneliness and optimism seem to have an interconnected relationship when considering older adults’ emotional well-being.

### Technological Advances

Technology and digital interventions have also surfaced to meet evolving emotional needs. Technological devices are designed to allow social interaction regardless of physical distance by texting, video chats, and new forms of social interaction, especially for older adults [12]. The best type of technological device (eg, desktop, phone, and tablet) is yet to be determined. In 1 systematic review, the use of computers and the internet in randomized controlled trials was not significantly related to lower levels of loneliness [13]; however, this area of the literature remains inconclusive.

Synchronous versus asynchronous technology is showing demonstrable conclusive differences. Synchronous tools, such as texting and videoconferencing, for example, have shown a 55% positive impact on significantly reducing loneliness [14]. Synchronous interventions, in general, have also been indicated as major contributing factors to general behavioral change. For instance, outside of loneliness examples, in 2 randomized control trials in which synchronous technologies (eg, telephone plus internet-based modules) were used, participants who were obese who attended at least 1 intervention session reported significant improvements in physical activity [15]. These studies highlight the efficiency of a synchronous experience.

Relatedly, computer-mediated communication has increasingly become a popular means to deliver health management interventions, including social-emotional services. Chatbots are a popular delivery and monitoring mode of such communication [16,17]. While trained, human moderators are typically required to complete some training to understand how and when to intervene within chats, chatbots use artificial intelligence (AI)–based models paired with natural language processing (NLP) to facilitate chats. Still, up to 86% of users prefer human moderators to chatbots [14,18,19]. Chatbots have been noted to increase frustration and skepticism among users and decrease the desired chat outcome due to their inflexibility [20,21]. In 1 study, users reduced purchase rates by >79.7% when chatting with a disclosed chatbot, even if an undisclosed chatbot was more effective than a human moderator [22]. Within social conversations, a lack of empathy, low understanding of contextual nuance, and bias in interactions have been noted [23]. Chatbots are typically designed to provide more definitive solutions, which may limit their sensitivity to non-preprogrammed topics such as crises or nuanced risk variables [24]. The use of ChatGPT-3–enabled chatbots, for example, has demonstrated the ability to reduce young adults’ self-reported scores on loneliness and suicidal ideation [25], yet there is inconclusive evidence that this pattern persists across studies [26]. ChatGPT has also been recommended to support older adults’, who experience mild cognitive impairment, experiences with loneliness and social isolation [27], but effectiveness data remain unavailable. In addition, concerns linger that chatbots’ lack of genuine empathy and emotional support may inadvertently lead to exposure to misleading or damaging content [28]. While chatbots continue to be trained to take on more human-like social cues within chats, trained human moderators continue to outperform chatbots in empathetic responses, with adequate sensitivity to understand crises and danger risks [21], without the accompanying privacy breach or security concerns [29]. Some chatbots have been noted to improve service efficiency, experience outcomes [16,17], be more cost-effective, and time-efficient; however, when users want social interaction, human interaction seems to outperform chatbots at this time [21,28].

Human moderators seem to be more effective at working with emotional and mental health topics within synchronous settings and can manage more nuanced situations than chatbots currently allow. For example, the often unrestricted and anonymous environments of web-based chats create spaces for antisocial behavior, such as bullying, web abuse, and harassment [30,31]. In 1 study, it was found that users were more engaged in chats when a human moderator was present, writing twice as many messages compared to unmoderated settings [32]. Users were also more likely to disclose negative emotions more openly with versus without a human moderator, and, when peers were present, peers took on the more supportive role in group settings with moderator support [32]. Human moderation also reduced the occurrence of harmful or toxic language 5-fold, from 5.2% of messages to only 1% [32]. Users who were in moderator-led chats also engaged in significantly more group problem-solving and coordination with one another, did not lose interest in the conversation, and the moderation positively encouraged coordination, supporting group cohesion and engagement [32]. Positive perspective changes, as noted by psycholinguistic factors (eg, focus-on-self, future- or past-focus, and positive or negative sentiment), were larger on average compared to unmoderated chats [32]. Finally, the moderated chats were found to stay significantly more on topic than unmoderated chats [32,33]. Such findings highlight that human-moderated chats in mental health settings support the users in many notable ways.

While synchronous technology and trained human moderators effectively impact positive change, the US health care system is experiencing a shortage of mental health care professionals to provide this support [34]. Given the effectiveness of synchronous technology in demonstrating significant sentiment and behavioral change and compounded by a shortage of mental health providers, there is a pressing need to explore innovative solutions to combat loneliness and foster optimism.

### Peer Support

The US President Joe Biden’s 2022 White House Brief referenced the mental health provider shortage with a call to action to “strengthen system capacity” [35] by elevating the role of peer support specialists and peer-based paraprofessionals. Peer support interventions combine persons with similar struggles or conditions to create an environment of mutual support [36]. In contrast to receiving treatment from a trained professional for a life challenge, peer support creates opportunities for connection, validation, and empathy around the shared experiences of living with a similar struggle. The “mutual empowerment” gained from a peer-led intervention has a different therapeutic benefit, allowing an individual more autonomy [37].

The type of peer support received is also important to consider. Informal peer support, or as referred to within the medical community, “patient-facilitated network,” is defined as support exchanged between people with similar life experiences [37]. Informal peer support can occur among people in a one-on-one conversation, group, or digitally and is not limited to any particular certification or training [38-43]. One randomized controlled trial that tested a peer-based intervention using a peer support listserve (unmoderated, unstructured, and anonymous) and a peer support bulletin found no significant differences between experimental and control groups’ outcomes of interest [38]; however, peer networks that used evidence-based strategies demonstrated greater success [42-44]. One such evidence-based approach is the use of motivational interviewing (MI) for inciting peer-supported change. The MI approach [45], which ascertains that for relational impact to occur, the conversation must apply the *spirit* of MI, which consists of demonstrating compassion, acceptance, evocation, and collaboration. The *spirit* of MI helps to foster a relationship that normalizes the peers’ struggle and positively reframes and adds compassion to the peer relationship. While there are a vast number of MI strategies, effective active listening skills such as open-ended questions, affirmations, reflective statements, summarizations, and information-giving are the most commonly used. The effectiveness of such MI strategies [41-44] has been linked with allowing conversations to progress from generic topics to engage the peer, deepen the focus of the conversation, evoke novel coping tools, and collaboratively identify plans for change. Pre-post studies have demonstrated improvements in several areas, including self-management [44], social cognition training [46], weight management [44,46,47], motivational improvements [42], psychoeducation [40,41], and parenting skills [39]. They have also shown reductions in psychiatric symptoms [40,41] and depression symptoms [42] as well as clinically significant improvements in cardiovascular symptoms [43]. Individual-reported symptoms also improve, including enhanced patient satisfaction [42,43], service user [47], knowledge [41,42], and psychosocial processes such as reduction in maladaptive social cognitions [46].

### Self-Determination Theory in Peer Support

The effectiveness of peer support interventions can be illuminated through the framework of self-determination theory, as delineated by Deci and Ryan [48] in 1985. According to this theory, 3 fundamental factors—autonomy, competence, and relatedness—facilitate individuals’ progression toward emotional change [48]. Autonomy pertains to an individual’s inclination to self-direct and exert control over their actions, while competence reflects the desire to acquire and master new skills before integrating them into behavior. Relatedness encompasses the need for interpersonal connections [49]. In peer support settings, as emotional struggles are processed, people may feel more autonomous because there is no mental health professional to serve as the expert guide, competence may be reinforced through mirroring and highlighting past coping skills, and participation in the peer community may inherently normalize the faced struggles because the person is connected to the community based on their unique struggle [50].

It is hypothesized that these dynamics within peer support settings positively correlate with improved mental health and well-being [50]. By harnessing the principles of autonomy, competence, and relatedness, peer support interventions offer a unique and potentially transformative and more accessible approach to addressing emotional difficulties and promoting individuals’ overall psychological resilience. With the rise of digital peer support, facilitated through various technological platforms, opportunities to extend the reach and impact of peer support interventions have expanded [51,52]. Digital peer support, including informal peer-to-peer networks, offers accessible avenues for individuals to connect and provide mutual assistance, thereby potentially mitigating feelings of loneliness and isolation [51].

Numerous studies have explored the effectiveness of digital peer support interventions, often in combination with evidence-based practices, in addressing mental health needs [37-44,46,47]. However, gaps remain in understanding the precise mechanisms through which these interventions influence emotional well-being and user engagement, particularly among the older adult population (aged ≥65 years).

Therefore, our study aims to fill these gaps by (1) accurately measuring changes in momentary loneliness and optimism experienced by older adults during participation in anonymous, informal, synchronous, and moderated digital peer support chats; and (2) investigating the specific impact of one-on-one (moderator+single user) versus group-based (moderator+≥2 users) anonymous, informal, synchronous, and moderated peer support chats on momentary loneliness trends, thus isolating the role of peers.

By pursuing these objectives, our study aims to contribute valuable knowledge to the field of digital peer support interventions among the ≥65 years age group, ultimately informing the development of effective strategies to address loneliness and enhance mental well-being among adults.

## Methods

### Supportiv Service Model

Supportiv is an anonymous, peer-to-peer service that provides mental, emotional, and social support through live moderated and synchronous small group chats available 24/7. It is accessible via links from health plans, employer portals, employee assistance programs, and other channels. Initiating with the prompt “What’s your struggle?” Supportiv uses AI-driven NLP to match users with others facing similar issues, creating groups of no more than 5 peers. Each group has a trained human moderator. Real-time availability of other users with similar struggles is necessary for users to enter a moderated small group chat. Users are autoselected, not self-selected, into a moderator+small group or moderator-only chat. Trained human moderators facilitate discussions and ensure a psychologically safe environment by enforcing chat rules and removing users for inappropriate behavior. They also lead problem-solving efforts. In cases of crisis, users are promptly directed to professional services, ensuring a comprehensive approach to support.

### Study Design

A retrospective observational study was conducted on user chats securely stored in the Supportiv database garnered between January and December 2022. Adults aged ≥65 years on a Medicare supplement plan were provided 1 year of no-cost access to the Supportiv service. From this sample, 699 peer support chats were included for further evaluation of inclusion criteria and subsequent analysis.

### Setting and Population in Study and Enrollment

Use of the Supportiv service was entirely voluntary. Due to the anonymous nature of the service, the user demographic information was unavailable other than the known variable of being in a cohort age-eligible for a Medicare supplemental plan. The initial evaluation of the user’s psychological history was forgone in an effort to expeditiously connect the user to a community with shared lived experiences. Every user was precision matched to a chat within 30 seconds of answering 1 question, “What’s your struggle?” The user’s response to this question was called the “user-chat-session-struggle” or, for short, the “user’s struggle.” Each chat session involved up to 4 users plus a moderator. Users, anonymously, joined informal, synchronous chats at variable times to discuss their struggles with a human moderator and up to 4 peers who shared a similar struggle.

During the synchronous chats, the human moderators used MI-based open-ended questions, affirmations, reflective statements, summarizations, and information-giving skills (eg, open-ended questions, affirmations, reflective statements, summarizations, and information-giving via web resources) to assist the user in exploring their struggle, focus the chat on the struggle, share topic-related resources, and invite peer collaboration to problem solve the users’ self-identified struggle.

After the chat session, each user may leave a comment about their experience. We manually compiled and categorized the comments.

### Analysis Inclusion Criteria

For each user chat, we used the score of the user’s struggle to evaluate the emotional intensity at the beginning of the session. On a scale of 1 to 10 (1=low, 5=moderate, and 10=high intensity), we set our threshold to be 5. Further description of the scoring is provided in the *Data Analysis* section. We excluded user chats when the struggle score did not surpass the minimum threshold to narrow the analysis to the user chats starting with at least moderate loneliness. The loneliness analysis included the users’ chats where the user-chat-session-struggle scored high on loneliness (eg, a score of >5 on a scale of 1-10). In other words, the “loneliness trend” represents the aggregate of all user chat sessions that started with a struggle and scored >5 for loneliness. We included all the user chat sessions in the optimism graphs.

Finally, during the propensity matching process, a user chat session was excluded from the analysis if a match was not found within the defined caliper. Further details about the propensity matching are provided in the *Moderator+Single User Chat Versus Moderator+Small Group Chat Emotion Trends* section.

### Data Analysis

#### Overview

Each user chat was treated separately, even if the same user participated in multiple chats, referred to as “user-chat-sessions” or, for short, “user chats.” Due to the anonymous nature of the platform, each user and their specific experience in a given chat session were considered independently, and data from different chats for the same user were not combined. This approach ensures that each user’s experience was analyzed in isolation, providing a more accurate and user-centric view of emotional changes. A user session was defined by the initial “struggle”—a brief and limited 300-character free-text response, plus all the messages exchanged in a chat. If the user joined another chat session, the user added a new struggle and began a new chat, which was processed separately as another independent session.

We independently quantified the changes in momentary loneliness and optimism of each user in the chat session. Each user’s message was processed in the context of its preceding messages from other users to capture their emotional journey throughout the chat session. The context was understood as concatenating all the preceding messages from other users in the same chat, starting from the latest message from the user of interest. The user’s struggle and the first message would not have a context and were evaluated with an empty context. For example, in the following conversation:

User A: message 1

User B: message 2

Moderator: message 3

User A: message 4

User B: message 5...

We used the assumption that message 1 and message 2 are the first messages of user A and user B, respectively, in the chat session. To quantify user A’s emotional journey, we evaluated message 1 with an empty context, and then, we evaluated message 4 in the context of message 2+message 3. Similarly, for user B, we evaluated message 2 without a context and message 5 in the context of message 3+message 4.

Each user’s message with its corresponding context was evaluated for loneliness using a scale of 1 to 10, where 1=low intensity of loneliness, 5=moderate intensity of loneliness, and 10=high intensity of loneliness. For the optimism model, the output is –1 for a negative sentiment, 0 for a neutral sentiment, and +1 for a positive sentiment. Our loneliness analysis specifically targeted chat sessions where participants exhibited at least moderate loneliness from the beginning. Therefore, we chose to view loneliness as a variable that can present varying intensity levels, while optimism is a variable with different states.

We performed two different analyses in this study: (1) loneliness and optimism trends across all user chat conversations and (2) isolating the role of peers in influencing emotional states through loneliness and optimism trends after propensity matching of chat with only 1 user and 1 moderator (moderator+single user chat) versus chats with ≥2 users and 1 moderator (moderator+small group chat).

#### Emotion Trends Across All User Chat Conversations

We calculated the average score in 1-minute intervals between 0 and 68 minutes (68 minutes is the 95% percentile of the conversation durations). We interpolated and extrapolated the scores of the user chats to find missing values in the range. During the first 5 minutes, the users describe and set up details about their struggles. Loneliness intensity during this time interval was extremely high (and optimism low), and thus, we used the 5-minute mark as the reference point for our change and statistical significance analysis to isolate the impact of the intervention and minimize inflation of the results.

In this analysis, we analyzed the change in emotion at the level of an individual user. Then, we combined it across all users to produce an aggregate graph for a specific emotion. We performed a 1-sided Mann-Whitney *U* test to compare the emotion scores at every time point with those at the beginning of the intervention (5-minute mark). A time point is deemed to exhibit significant changes if *P*<.05.

#### Moderator+Single User Chat Versus Moderator+Small Group Chat Emotion Trends

This analysis compares peers’ impact in the chats. We used propensity matching to create 2 separate but equal comparison cohorts: moderator+single user and moderator+small group chats, and we used the same aggregation methodology to create the emotion trends. If a user on the Supportiv service chatted with ≥2 peers and the moderator, they were included in the moderator+small group chat. However, if the user only chatted with the moderator, they were included in the moderator+single user chat cohort.

After splitting the user chat conversations into 2 cohorts, we matched the users in the 2 cohorts by training a logistic regression classifier with the following features: user-chat-session topic, device type used, and emotional intensity of the specific emotion of the user struggle to ensure high alignment at the beginning of the chat between the users in 2 cohorts. The emotional intensities were quantified on a scale of 1 to 10 for loneliness and positive, neutral, and negative for optimism. The logistic regression classifier predicts the propensity (likelihood) of each user-chat-session belonging to a cohort. The model’s inferences are commonly referred to as propensity scores. We used 25% of the SD of all propensity values as our caliper (radius of search) for matching. For every user chat in the moderator+single user chat cohort, we found the closest match in the moderator+small group chat cohort using a k-nearest neighbor classifier that considers within the caliper.

We performed propensity matching independently for each trend under consideration. For example, to plot the loneliness (optimism) trend, we aligned the intensity of loneliness (optimism), the user’s chat session topic, and device type between the 2 cohorts. The topics used for the user’s chat struggles were physical health or medical, social connection, loneliness, anxiety, mental health conditions, excluding anxiety and depression, trauma, depression, caregiving or parenting, stress, identity, finances, productivity or work, suicide or self-harm, and others. These topics were chosen after manually inspecting the chats and drawing general themes across the population. Due to the platform’s anonymity, individual users’ demographic data are unavailable; therefore, propensity matching based on demographic data is not feasible.

In addition to the statistical test to find when each trend exhibited a statistically significant change (in reference to the 5-minute mark), we performed a 1-sided Mann-Whitney *U* test to detect when the moderator+small group chats outperformed the moderator+single user chat ones. We highlighted when there was a consistently significant difference between the 2 trends (*P*<.05).

### AI Emotion Models

Our analysis used a third-party, public NLP model from OpenAI called GPT-4. The model assigned a score to each user message and its preceding context for loneliness and optimism. We used a few-shot learning approach where the model saw 10 examples indicating different loneliness intensities and optimism to help calibrate the model’s interpretations [53]. GPT-4 displays an advanced capacity to interpret emotion, surpassing benchmarks from the general population and offering a remarkably congruent performance in emotion interpretation [21]. Textboxes 1 and 2 demonstrate the examples we provided GPT-4 to predict loneliness and optimism levels, respectively.

**Examples provided to GPT-4 to interpret loneliness intensities in a few-shot manner.**

**Intensities and loneliness**

- 1=low
  “Take care everyone. Thanks for the great talk.”
- 5=moderate
  “I went on Monday and today. After today’s class is when I felt worse as I chatted with a lady in class who told me that she got really depressed when she retired.”
- 10=high
  “I just always feel totally alone. Like no one understands what is going on in my head.”

**Examples GPT-4 used to interpret optimism intensities in a few-shot setup.**

**Intensities and optimism**

- 1=positive
  “I am optimistic that I might slowly get better.”
- 0=neutral
  “I didn’t do much today. Just stayed at home and watched some TV.”
–1=negative
  “Feeling so overwhelmed by everything. It’s hard to see a way out right now.”

In addition, we evaluated the performance of GPT-4 in quantifying loneliness and optimism with our few-shot prompt on publicly available datasets. GPT-4 exhibits promising performance on these datasets, comparable to or superior to state-of-the-art models [54-56]. Multimedia Appendix 1 provides more details on this analysis. Furthermore, we manually inspected 25% (>2500 examples) of GPT-4’s interpretations of the loneliness and optimism intensities to confirm their validity and establish further confidence in GPT-4’s interpretations.

Similarly, we used GPT-4 to classify the user chat session topics for propensity matching. We concatenated all the user messages in the chat session to capture the user chat session topic. Furthermore, we manually confirmed the correctness of the model’s interpretation of all user session topics.

### Ethical Considerations

This case study was conducted retrospectively on an anonymous dataset and was deemed exempt from human ethics research review by the Alpha Institutional Review Board (IRBSPE001A).

## Results

### Overview

**Figure 1:**
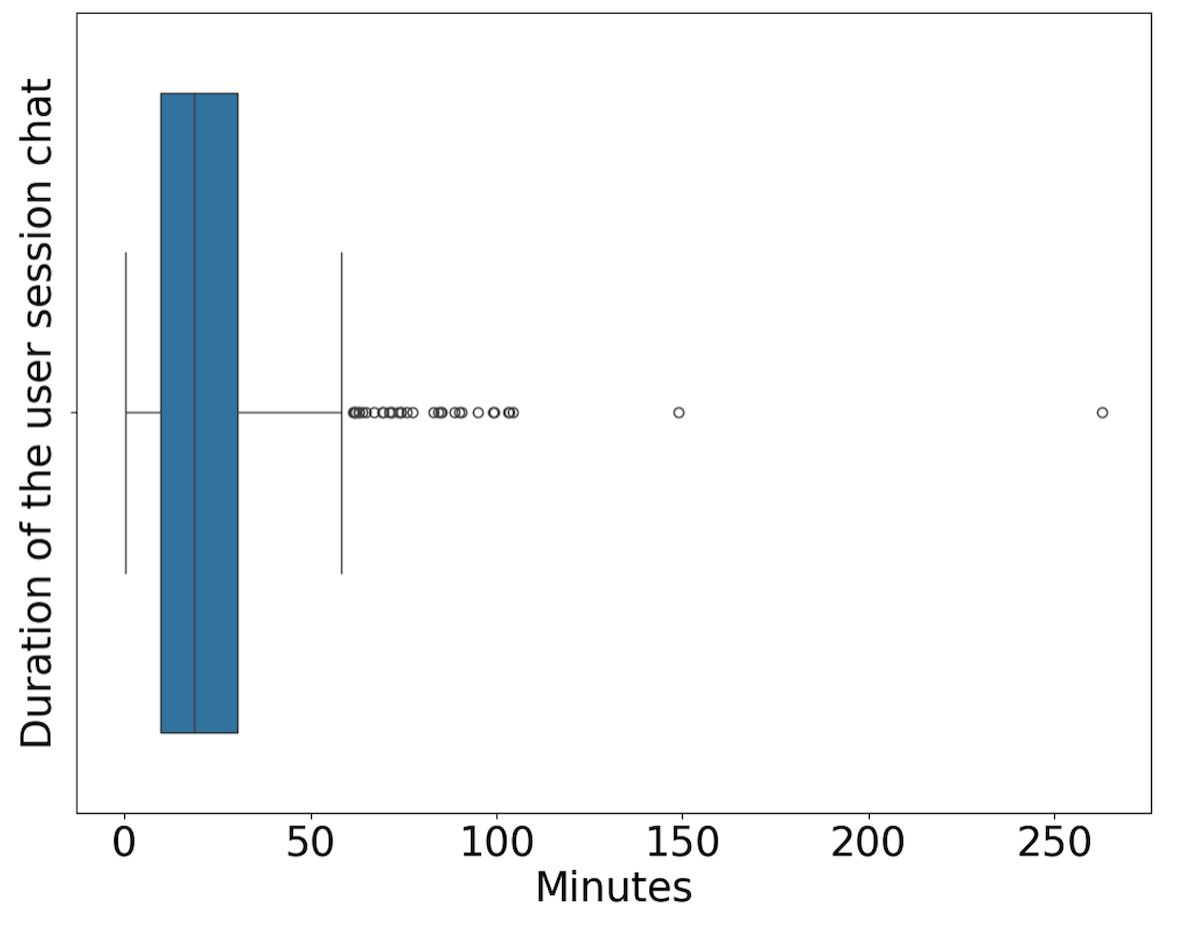
Duration of the user session chat for all 699 participants, with an average of 19.4 minutes and a minimum of 0 and maximum of 262 minutes in moderated digital peer support chat.

Participants completed 699 digital peer support chats and spent an average of 19.4 minutes interacting with a peer group. Figure 1 displays the duration of the user session chat for all participants, with a minimum of 0 and a maximum of 262 minutes.

The most common struggle topic expressed by users was related to physical health or medical (n=98, 20%), followed by social connection (n=73, 15%), loneliness (n=43, 9%), anxiety (n=43, 9%), depression (n=43, 9%), trauma (n=34, 7%), stress (n=34, 7%), caregiving or parenting (n=29, 6%), mental health conditions (n=24, 5%), financial (n=19, 4%), identity (n=10, 2%), productivity or work (n=5, 1%), suicide or self-harm (n=5, 1%), and the remaining 6% (n=29) were dispersed and categorized under the other topic. Most of the users used their desktop (n=269, 55%), followed by mobile phones (n=205, 42%) and tablets (n=15, 3%).

Of all 699 user-chat-sessions, 489 (70%) included a message in the room. Note that 210 (30%) users who dropped off without sending messages in their chat sessions cannot be analyzed further for emotional trends because the only available data point is the user struggle, and we cannot infer how their emotion intensities change without any messages. The loneliness analysis included 180 (25.8%) user chat sessions. The discrepancy between the total number of user-chat-sessions considered (n=489) and those included for loneliness (n=180) arose from the minimum threshold inclusion criteria, wherein a minimum of a “5” loneliness score was necessary at the beginning of the conversation.

**Figure 2:**
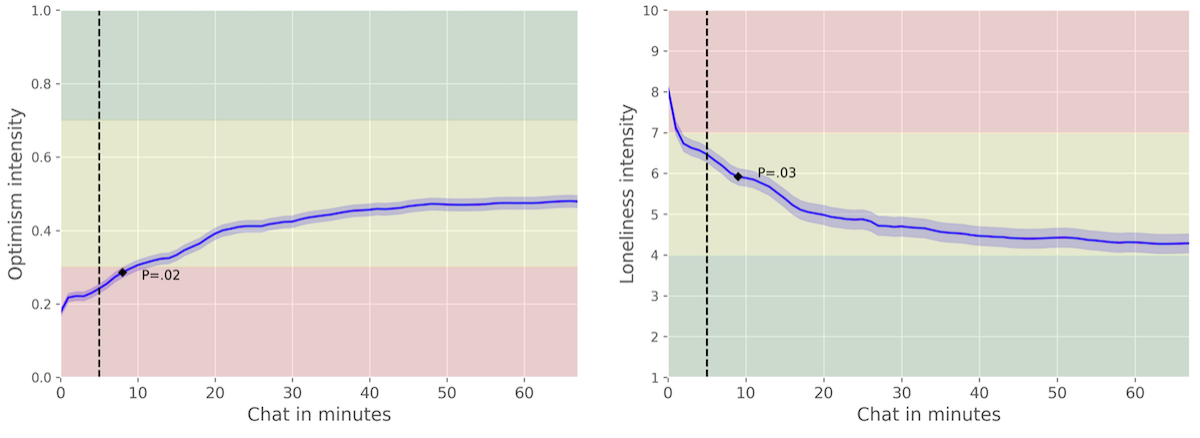
Emotion trends during chat sessions (for users included in the aggregate analysis). We observed significant changes at the following time stamp and beyond: >8 minutes for optimism and >9 minutes for loneliness. The green area (1-4) signifies “low” levels of loneliness, while yellow (4-7) and red (7-10) show “moderate” and “high” levels of momentary loneliness, respectively. The optimism values <0.3 are considered “negative,” >0.7 “positive,” and between 0.3 and 0.7 “neutral.” The 5-minute mark, highlighted with a dotted line, is the reference point for the statistical tests and change calculations.

Across all user chat sessions, there was an average improvement in optimism of 49.43% and a corresponding decrease of 33.71% in momentary loneliness. All observed changes were statistically significant compared to 5 minutes (Table 1), especially after 8 minutes; we observed a significant increase in optimism, and after 9 minutes, we observed a significant drop in momentary loneliness. Figure 2 depicts the trends of loneliness and optimism with the significant time points highlighted. The optimism values started at 0.24 (SD 0.27) at the beginning of the intervention (5-minute mark), well into the negative, and rose to 0.48 (SD 0.36) in the neutral zone. The loneliness intensities started at 6.47 (SD 2.53; at the 5-minute mark) in the top of the moderate zone and declined to 4.29 (SD 3.11) toward the low bounds of the moderate range.

Table 1 summarizes the emotion trends’ starting value, ending value, percentage change, significant time point, and number of user chat conversations for loneliness and optimism trends.

Supportiv is a web-based platform; thus, the digital proficiency divide may prevent some older adults from accessing and benefiting from it. Supportiv will continue implementing strategies to broaden outreach and user engagement, especially among those less familiar with digital platforms. These strategies include an intuitive, straightforward interface, adjustable font size, etc.

**Table 1:** Statistical analysis of emotion score changes for users.

Emotion	User chat sessions, n	5 min, mean score (SD)	68 min, mean score (SD)	Change at 68 min (%)	Change at 19.4 min^a^ (%)	Time of significant change (min)	*P* value
Optimism	489	0.24 (0.27)	0.48 (0.36)	49.43	36.11	>8	.02
Loneliness	180	6.47 (2.53)	4.29 (3.11)	33.71	22.40	>9	.03

^a^The average chat session duration is 19.4 minutes.

### Future Directions and Work

Investigating the mechanisms through which chat-based support facilitates emotional improvement is critical to guide ongoing inquiry in this field. Plans for future studies include expanding to derivative descriptions of each emotion to explore how optimism changes over time in the chat and measuring the interactions between different emotions. In addition, a granular analysis of the impact of the applied MI strategies on emotion change described in this paper will be reviewed for their direct impact in this digital setting. Finally, we will analyze for a larger sample size and other populations.

**Figure 3:**
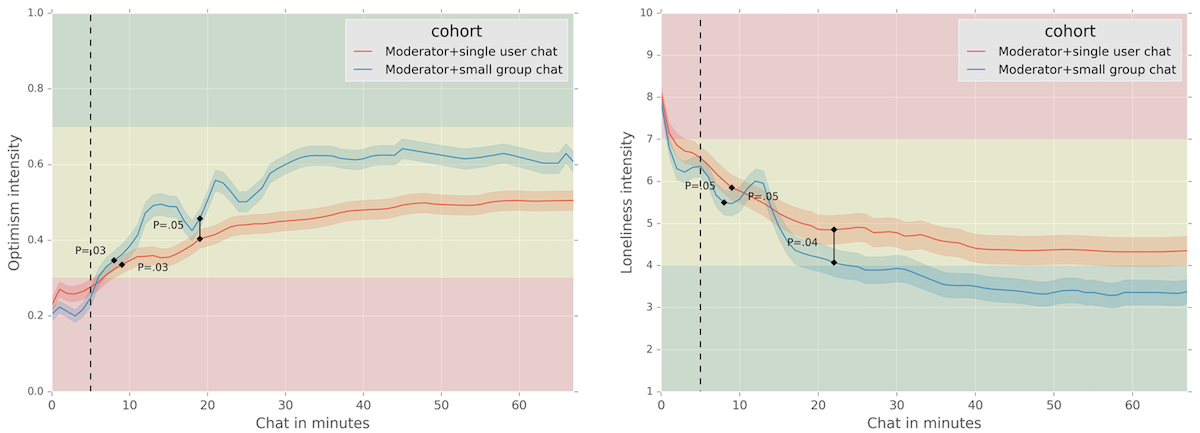
Emotion trends during chat sessions for moderator+single user chat vs. moderator+small group chat cohorts. We observe significant changes >8 minutes for moderator+small group chat cohort and >9 minutes for moderator+single user chat cohort for both loneliness and optimism trends. The 5-minute mark, highlighted with a dotted line, is the reference point for the statistical tests and change calculations. The green area (1-4) signifies “low” levels of loneliness, while yellow (4-7) and red (7-10) show “moderate” and “high” levels of momentary loneliness, respectively. The optimism values below 0.3 are considered “negative,” above 0.7 “positive,” and between 0.3 and 0.7 “neutral.” We observe a significant difference between the moderator+single user chat and moderator+small group chat cohorts, with moderator+small group chat cohorts outperforming after 19 and 21 minutes for optimism and loneliness, respectively.

Multimedia Appendices 1 and 2 outline the details of the propensity score matching. Figure 3 demonstrates how the matched moderator+single user chat emotion trends compare to moderator+small group chats. While all trends exhibit significant changes, the loneliness and optimism moderator+small group chat trends significantly improve over the moderator+single user chat chats. The graphs mark when the changes between the moderator+single user chat and moderator+small group chat trends become statistically significant (*P*<.05). For each trend, the time stamp where the changes were significant is highlighted. Specifically, the moderator+small group chat cohorts significantly outperform the moderator+single user chat cohort at >19 (*P*=.049) and >21 (*P*=.04) minutes for optimism and loneliness, respectively.

Table 2 depicts the number of user chat sessions included in each trend. Only a subset of the user-chat-sessions met the inclusion criteria for loneliness, that is, started with a struggle that scored higher than the set threshold of 5 and hence the discrepancy between the number of user chats included in loneliness in Table 2 and those after propensity matching (Multimedia Appendix 2).

In moderator+single user chats, we observed an increase of 45.22% in optimism and a drop of 33.63% in momentary loneliness. In moderator+small group chats, however, we observed a much more pronounced increase of 58.9% in optimism and a decline of 45.3% in momentary loneliness. The optimism trend for moderator+single user chats starts at 0.28 (SD 0.28) and ends at 0.51 (SD 0.35), and the moderator+small group chats’ optimism trend starts at 0.25 (SD 0.32) and ends at 0.61 (SD 0.35). The momentary loneliness trend for moderator+single user chats starts at 6.56 (SD 2.54) and drops to 4.35 (SD 3.15), and the moderator+small group chats’ loneliness trend starts at 6.27 (SD 2.32) and drops to 3.38 (SD 2.73). In addition, the significant difference in time in the trends for moderator+small group chats is a minute less than that for moderator+single user chats (8 minutes vs 9 minutes) for optimism and loneliness trends. Finally, the SD values for the moderator+small group chat trends were consistently lower than the ones for the moderator+single user chat cohort, indicating a more consistent decline in momentary loneliness, accompanied by a rise in optimism in moderator+small group chats compared to the moderator+single user chat chats.

Textbox 3 illustrates some central themes from users’ comments throughout the digital chats, including positive affirmations of the experiences and concerns around privacy and barriers to accessing mental health services.

**Table 2:** Statistical analysis of emotion score changes for moderator+single user chat versus moderator+small group chats.

Emotions	Moderator+single user chat							Moderator+small group chat					
	Values, n	5 min, mean (SD)	68 min, mean (SD)	Change (%)	Time of significant change (min)	*P* value	Values, N		5 min, mean (SD)	68 min, mean (SD)	Change (%)	Time of significant change (min)	*P* value
Optimism^a^	188	0.28 (0.28)	0.51 (0.35)	45.22	>9	.03	188		0.25 (0.32)	0.61 (0.35)	58.9	>8	.03
Loneliness^a^	86	6.56 (2.54)	4.35 (3.15)	33.63	>9	.047	94		6.27 (2.32)	3.38 (2.73)	45.3	>8	.046

^a^Moderator+small group chats significantly outperform the moderator+single user chats.

**Selected user comments and associated themes.**

**Comments**

- Privacy
  - “Is [my health plan] going to see our chat?...I wish I could give you a hug.”
  - “How does this work and is the info shielded from my health insurance.”
- Loneliness
  - “Thank you for helping me. I don’t feel quite as alone and hopeless.”
  - “Here is kind of like having a friend...It occurs to me that I could talk with someone here I just want to connect with someone who will care.”
  - “I feel better saying how I feel. Nobody wants to hear it. But it clears my mind.”
  - “We are mainly a “senior” population and people do not like to make waves. Sometimes people call me asking for guidance on issues here, but I no longer know who I can trust... Again, thank you for listening.”
- Mental health care access
  - “Everyone says to get help. Well, try to find help that 1) takes insurance and 2) has openings”
  - “Few therapists take insurance and of those who do the good ones are filled up and the [ones] who have openings have openings for a reason”
  - “I have a psychologist. She is helpful, but she doesn’t take insurance, so I can’t go as often as I need.”

## Discussion

### Principal Findings

Among this cohort of older adults, using an informal, synchronous, and moderated digital peer support service was associated with reduced momentary loneliness and a corresponding improvement in optimism. Consistent with the quantitative language processing analysis, many users’ comments centered around loneliness. These results are promising, considering the need for effective loneliness interventions among older adults.

Loneliness is a qualitative variable reflecting one’s subjective perception of their level of closeness to others. Theoretical frameworks describe 2 distinct experiences of loneliness, one that is experienced as a fleeting or momentary emotion and another where the emotion is far more persistent and intractable [57]. Given this and other research on peer support, a brief, anonymous digital peer support intervention is likely to be impactful for those who tend to have momentary loneliness, but the impact on persistent loneliness is outside the scope of this study. Users with momentary loneliness could find digital peer support a valuable tool in combating their loneliness. A meta-analysis of interventions for loneliness cited the creation of additional opportunities for social interaction as a critical strategy, and those targeting “maladaptive social cognition” appear to be the most effective [57]. Supportiv provides access, psychological safety, and opportunity for social interaction. Moderators trained to provide psychological and digital safety help amplify environmental access with the addition of safe social support. In the small group format, peers are no longer alone, which is most opportune for changing an individual’s perception of momentary loneliness [57,58] and building on the protective factor of optimism [9]. This study shows that chatting with a small group can accelerate the improvement in momentary loneliness and optimism trends compared to 1 person (moderator) alone.

Trained human moderators are a key component to the findings. While chatbots are available on the market to address emotional concerns in rule-based, semi–rule-based, or free-chat formats, emerging literature has indicated that chatbots often lack the contextual understanding, personalization, and appropriate crisis responses within text-based interactions [59]. Chatbots may apply a one-size-fits-all approach to addressing emotional concerns, such as sharing a joke to lighten the mood without considering the context. They may also struggle with understanding detailed user input or unrelated responses and might either overuse or underuse crisis protocols when needed [59]. Trained, human moderators can quickly build trust with the user by understanding the nuanced needs of the user while also shifting to personalized responses and, with training, evaluate and use appropriate crisis protocols and MI strategies. These key features ensure psychological safety and trust for the user.

Study results also present supplementary data regarding “relatedness” as cited within “self-determination theory” among older adults. Relatedness, which is best described as a person’s desire for interpersonal connections [50], is both an inherent human desire and serves to normalize emotional struggles via acceptance from another [50]. Our results indicate that the reduction in momentary loneliness may be best explained through the perceived “relatedness” experienced by users and may be possible as quickly as 9 minutes within a moderator+single user chat and 8 minutes in a moderator+peers chat. While relatedness data among older adults are understudied in similar contexts, social media text analysis presents the nearest comparison. Social media (eg, Facebook, Reddit, Instagram, and TikTok, etc) research, wherein large text-based data are evaluated through machine learning techniques [60] to recognize patterns in psychiatric [60] and crisis detection, presents the possibility of providing an “objective” snapshot of relatedness techniques. Our methodology and corresponding results have delivered the “objective” snapshot in relation to momentary loneliness and optimism among older adults within a digital context. Future studies may now be able to delve deeper into understanding the causes behind this change to better leverage relatedness.

Furthermore, in a controlled setup, we observed that the moderator+small group chats offer significant improvements compared to moderator+single user chat interventions, further reinforcing the promise of a peer support solution for dealing with momentary loneliness among older adults. The benefit of this peer effect on momentary loneliness becomes even more critical among older adults, as a digital offering directly removes many known barriers for this population [61]. For example, limited financial or transportation resources often prevent older adults from socializing in person [61]. Others may be too medically vulnerable to participate in community gatherings [61]. However, these obstacles can be overcome when socialization occurs digitally. This benefit, of course, depends on the digital accessibility and literacy of the user. Though historical convention has held that older adults will not or cannot use digital technology, the events of the global COVID-19 pandemic as well as ongoing change in our social landscape have produced a much more heterogeneous older adult population with regard to ownership and use of technology. In this study and consistent with the existing literature, desktop computer was the most popular device among the older adults’ sampled and allowed access to Supportiv’s service for improvements to their mental well-being [62]. Additional high-quality studies are necessary to build on this finding. However, there are still notable differences in technology and internet use based on race, ethnicity, and income level [62,63]. The “digital divide,” which refers to the gap between those older adults who have access to the internet, technological resources, and training to build their digital skill set, remains a major barrier to equitable access [64]. Lack of reliable access to technology and the corresponding digital literacy to use technology remain a significant concern and present a systemic barrier to a solely digitally based peer experience addressing loneliness. Relatedly, negative effects of technology have been noted. For example, persistent technological exposure has been connected with attention concerns across all age groups [65], sleep quality concerns that in turn increase risk for Alzheimer disease among older adults [66], and increased perceived social isolation with prolonged (≥2 hours daily) versus brief exposure (≤30 minutes daily) [67,68]. The latter concern is hypothesized to occur due to a reduced exposure to real-world social interactions [67]. Still, given the study results suggesting that momentary loneliness can be shifted within 8 to 9 minutes, brief exposure to digital peer support appears to be a worthwhile opportunity.

Another barrier to the participation of older adults in web-based and digital supports includes the need to ensure privacy. Privacy was queried explicitly in some of the user’s comments, but the extent of the concern across the entire cohort of users is difficult to assess. The anonymous nature of the Supportiv service directly addresses privacy concerns, as users have to share only their struggles and are not asked for any personally identifiable information. Anonymity is an intentional feature of Supportiv’s service, as it has been shown through research to increase sharing of topics of a sensitive nature [69]. Anonymous digital experiences have positively impacted areas outside of well-being, including in educational and employer settings, for supporting advocacy and system navigation [70,71]. The addition of a moderator trained in evidence-based practices to ensure psychological safety allows for directed service referrals that address the users’ individualized underlying needs in real time.

An additional theme in the comments was the challenge of older adults in finding and paying for mental health care. The rates of depression and suicide are higher among lonely older adults [72]. This highlights the importance of having alternative pathways to support older adults experiencing challenges in accessing mental health care. The impact of peer support is most clearly established in mental health [72-75]. This presents an area of opportunity for expanding older adults’ awareness of low-cost, easily accessible, and nonstigmatizing digital alternatives such as Supportiv, which have no dependency on access to licensed professionals. If access to mental health services is altogether insufficient, there must be the pursuit of opportunities that address mental health needs in real time.

### Study Limitations

Study limitations are directly related to real-world evidence generation. The service is designed to avoid diagnosis and treatment, focusing primarily on enhancing peer-based social support. The user journey is anonymous and intentionally built to avoid lengthy preassessments that delay peer engagement. This presents challenges to measuring the longitudinal impact of the intervention, especially among repeated service users. The anonymity prevents comparison of repeat users’ demographics and the trends of emotional state before, in between, and upon completion of overall engagement with the Supportiv service. Moreover, anonymity interfered with controlling for factors such as marital status, demographic information, and technical savviness. The aggregate loneliness and optimism trends cannot control for the random effects of the same user visiting the platform repeatedly.

This study assumes that the AI model suffers from minimal systemic biases. The study’s aggregate nature cancels noise in individual user-chat-sessions, leaving the aggregate trends noise free. Systemic biases in the model’s interpretation can negatively affect the results presented in this study. However, due to the model’s high performance, we expect minimal systemic biases for the emotions [21].

We recognize the impact of selection bias, where those drawn to this service might already be more inclined and ready to engage in self-care or improvement. Those who may not be ready and/or did not benefit from the service may have exited the chat early, but similar to therapy attrition, this is difficult to methodologically decipher. In addition, this study was limited to those with digital access. Still, it remains especially important to consider who is not represented in this cohort due to emotional health stigma and disparities in digital literacy and digital access. During this analysis, Supportiv services were only available in English, so it is difficult to assess the value for persons whose primary language is not English.

### Practical Considerations

We use state-of-the-art security measures (such as at-rest and in-transit encryption) to minimize potential privacy and security risks in handling users’ data. In addition, to ensure user safety, Supportiv takes utmost care in identifying and referring users in crisis. All moderators undergo extensive training in identifying crises, are taught a subclinical protocol, and provide external referrals for any reported need for professional services. They are also regularly monitored for their handling of crises and additional service delivery quality.

### Conclusions

In conclusion, a chat-based peer support service may be a viable intervention to help address momentary loneliness in older adults as an alternative to traditional care. These promising results positively contribute to further study in this area. Bringing together groups of older adults who are experiencing loneliness and those with shared experiences creates an environment where individuals immediately feel less alone. Few models can so elegantly deliver opportunities to change one’s perception of momentary loneliness in real time.

## Appendix

Multimedia Appendix 1 Performance evaluation of GPT-4 in quantifying loneliness and optimism with our few-shot prompt on publicly available datasets.

Multimedia Appendix 2 Search caliper, samples in each cohort after the propensity matching, and the maximum standardized mean differences (Cohen d) after matching.

## Abbreviations

AI: artificial intelligence
MI: motivational interviewing
NLP: natural language processing

## Data Availability

The data sets generated and analyzed during this study are not publicly available due to their proprietary nature but may be available from Supportiv upon reasonable request. Please contact info@supportiv.com.
