# Supplementary material for: Role of Synchronous, Moderated, and Anonymous Peer Support Chats on Reducing Momentary Loneliness in Older Adults: Retrospective Observational Study
Source: JMIR Form Res. 2024 Oct 25;8:e59501. doi: 10.2196/59501 (PMC11549579; doi:10.2196/59501)
Supplement: Multimedia Appendix 1 [file formative_v8i1e59501_app1.docx]

## Appendix 1

Jiang et al. released the FIne-Grained Loneliness (FIG-Loneliness) dataset and trained a few supervised NLP models to detect loneliness [1]. We used this dataset with a few-shot learning prompt to evaluate the performance of GPT-4. The few-shot GPT-4 model outperformed Jiang et al.’s baseline supervised LSTM model. Table S1 shows the results for few-shot GPT-4 and how it compares to the models published by the authors. The results show that GPT-4 has a promising 93% F1 score in detecting loneliness.

| **Emotion** | **Model** | **Accuracy** | **Binary Precision** | **BinaryRecall** | **BinaryF1** |
| --- | --- | --- | --- | --- | --- |
| **Loneliness** | **Supervised LSTM*** | 0.90 | 0.89 | 0.9 | 0.89 |
|  | **Supervised HDLN*** | 0.98 | 0.96 | 0.99 | 0.97 |
|  | **Few-Shot GPT-4** | 0.93 | 0.94 | 0.91 | 0.93 |

Table S1: The few-shot GPT-4 outperforms the baseline supervised LSTM in quantifying loneliness. However, the supervised HDLN model performs the best. The asterisk highlights the results published by the creators of the FIG-Loneliness dataset’s creator.

It is worth emphasizing that supervised models “study” examples of similar data (training dataset) during their training process. In contrast, few-shot learning models only see a few examples from the training dataset in their context. The models in [1] were trained on 59k samples, while the few-shot GPT-4 only sees ten examples in its context, five positive and five negative. The high performance of the Supervised HDLN is not a surprise, and despite only having access to 10 examples, GPT-4’s loneliness quantification capabilities are impressive.

Additionally, Google released GoEmotion: A Dataset of Fine-Grained Emotions [2]. This dataset includes an optimism label. Google’s best supervised Bidirectional Encoder Representations from Transformers (BERT) model published in this study did much worse than the few-shot GPT-4 results. The BERT model in [2] was trained on 46k samples, while the few-shot GPT-4 only “studies” ten examples in its context, five positive and five negative.

Table S2 below shows the results for few-shot GPT-4 and how it compares to the models published by the authors [2]. The results show that few-shot GPT-4 outperformed the supervised BERT model in detecting optimism.

| **Emotion** | **Model** | **Accuracy** | **Micro Precision** | **Micro Recall** | **Micro F1** |
| --- | --- | --- | --- | --- | --- |
| **Optimism** | **Supervised BERT*** | Not Reported | 0.41 | 0.69 | 0.51 |
|  | **Few-Shot GPT-4** | 0.78 | 0.78 | 0.78 | 0.78 |

Table S2: The few-shot GPT-4 outperforms the supervised BERT model in quantifying optimism. The asterisk highlights the results published by the GoEmotion dataset’s creators.

The prompts used for loneliness and optimism evaluations are similar to the ones we used in our data processing pipeline, with a slight difference. In the manuscript's data processing pipeline, we instruct GPT-4 to determine the intensity of the emotion. Unfortunately, there are no publicly available datasets with different intensities of loneliness or optimism; thus, we present the best evaluation of GPT-4’s performance with the publicly available datasets for loneliness and optimism.

To evaluate the performance of GPT-4 on a dataset with multiple intensities, we considered Sentiment140 datasets [3] because optimism is closely tied to the sentiment in the sentences. The Sentiment140 dataset is gathered from Twitter by the researchers at Stanford [3] and includes three intensities of “negative,” “neutral,” and “positive” sentiments. Table S1 demonstrates the performance of a few-shot GPT-4 compared to the model Sahni et al. developed in their paper. The results show that few-shot GPT-4 outperformed the supervised Effective Word Score model (EWS) in detecting sentiment.

| **Emotion** | **Model** | **Accuracy** | **Micro Precision** | **Micro Recall** | **Micro F1** |
| --- | --- | --- | --- | --- | --- |
| **Sentiment** | **Supervised Effective Word Score (EWS)*** | 0.85 | Not Reported | Not Reported | Not Reported |
|  | **Few-Shot GPT-4** | 0.87 | 0.87 | 0.87 | 0.87 |

Table S3: The few-shot GPT-4 outperforms the supervised EWS model in quantifying sentiment. The asterisk highlights the results published by the Sentiment140 dataset’s creators.

In conclusion, GPT-4’s performance in quantifying loneliness and optimism is superior to or on par with other state-of-the-art natural language models.

##

**References**

1. Jiang Y, Jiang Y, Leqi L, Winkielman P. View of many ways to be lonely: fine-grained characterization of loneliness and its potential changes in COVID-19. In: Proceedings of the Sixteenth International AAAI Conference on Web and Social Media; 2022.
2. Demszky D, Movshovitz-Attias D, Ko J, Cowen A, Nemade G, Ravi S. GoEmotions: a dataset of fine-grained emotions. In: Proceedings of the 58th Annual Meeting of the Association for Computational Linguistics. Association for Computational Linguistics; 2020. Accessed October 17, 2024.<http://dx.doi.org/10.18653/v1/2020.acl-main.372>
3. Sahni T, Chandak C, Chedeti NR, Singh M. Efficient Twitter sentiment classification using subjective distant supervision. In: 2017 9th International Conference on Communication Systems and Networks (COMSNETS). IEEE; 2017. Accessed October 17, 2024.<http://dx.doi.org/10.1109/comsnets.2017.7945451>
