# Supplementary material for: Role of Synchronous, Moderated, and Anonymous Peer Support Chats on Reducing Momentary Loneliness in Older Adults: Retrospective Observational Study
Source: JMIR Form Res. 2024 Oct 25;8:e59501. doi: 10.2196/59501 (PMC11549579; doi:10.2196/59501)
Supplement: Multimedia Appendix 2 [file formative_v8i1e59501_app2.docx]

## Appendix 2

Table S1 shows the search caliper, samples in each cohort after the propensity matching, and the maximum standardized mean differences (Cohen's D) after matching. The search caliper was chosen based on the 25% standard deviation of propensity scores. Cohen's D is a common way to measure effect size, i.e., how significant an effect is. After matching, a lower Cohen’s D value indicates a better alignment between the two cohorts. After matching, all the Cohen’s D values fall below 0.22, indicating a small effect size and good alignment between the two cohorts. The number of user chat sessions is lower because some user-chat-sessions in the moderator+single user chat cohort did not match a session in the group-chat cohort within the caliper radius.

Table S1: The search caliper, number, and maximum Cohen’s D of user chat sessions post propensity matching for loneliness and optimism.

| **Emotion** | **Caliper** | **Number of matched user-chat sessions** | **Maximum Cohen’s D** |
| --- | --- | --- | --- |
| Optimism | 0.040 | 188 | 0.09 |
| Loneliness | 0.041 | 201 | 0.22 |

|  | |
| --- | --- |
|  | |
|  | |
|  | |

## 
